# Cursorial ecomorphology and temporal patterns in theropod dinosaur evolution during the mid-Cretaceous

**DOI:** 10.1098/rsos.241178

**Published:** 2025-01-15

**Authors:** Kohta Kubo, Yoshitsugu Kobayashi

**Affiliations:** ^1^ Department of Natural History Sciences, Hokkaido University, Kita 10, Nishi 8, Kita-ku, Sapporo, Hokkaido 060-0810, Japan; ^2^ Department of Earth and Planetary Science, Graduate School of Science, The University of Tokyo, 7-3-1, Hongo, Bunkyo-ku, Tokyo 113-0033, Japan; ^3^ Hokkaido University Museum, Kita 10, Nishi 8, Kita-ku, Sapporo, Hokkaido 060-0810, Japan

**Keywords:** Coelurosauria, mid-Cretaceous, arctometatarsus

## Abstract

Coelurosauria, including modern birds, represents a successful group of theropod dinosaurs that established a high taxonomic diversity and significant morphological modifications. In the evolutionary history of this group, a specialized foot morphology, the arctometatarsus, evolved independently in several lineages and has been considered an adaptation for cursoriality. While its functional significance has been extensively studied, the temporal pattern of this parallel evolution, as well as its origin and influencing factors, remains largely unresolved. Here, we show the temporal evolution of cursorial traits, including the arctometatarsus and hind limb proportions. Our study reveals that the proportional elongation of distal hind limb segments preceded the evolution of the arctometatarsus in ornithomimosaurs and oviraptorosaurs. In contrast, in tyrannosauroids, alvarezsaurs and troodontids, the proportional elongation of the tibia and metatarsals occurred in parallel with the acquisition of the arctometatarsus. The evolutionary history of the arctometatarsus further highlights the presence of a phylogenetic constraint outside Coelurosauria, as this foot specialization is restricted to members of this group. Finally, our date estimation, based on compiled evolutionary patterns, demonstrates that these cursorial traits emerged during the mid-Cretaceous (93–120 Ma), suggesting selection on theropod locomotor performance throughout this interval.

## 1. Introduction

Coelurosauria is a group of theropod dinosaurs that achieved remarkable taxonomic and morphological diversity along with wide geographical distribution [1,2]. They occupied a variety of ecological niches, serving both as predators and prey in the Cretaceous terrestrial ecosystems [3–5]. Coelurosaurs are also known for morphological adaptations leading to avian characteristics such as the feather [6,7], tuck-in resting pose [8,9], edentulous skull [10,11] and rapid growth rate [12]. The evolutionary history of coelurosaurs further documents the convergent evolution of several ecological traits, including body size [3,12,13], herbivorous diets [10,14–16] and flight ability [17].

**Figure 1.**
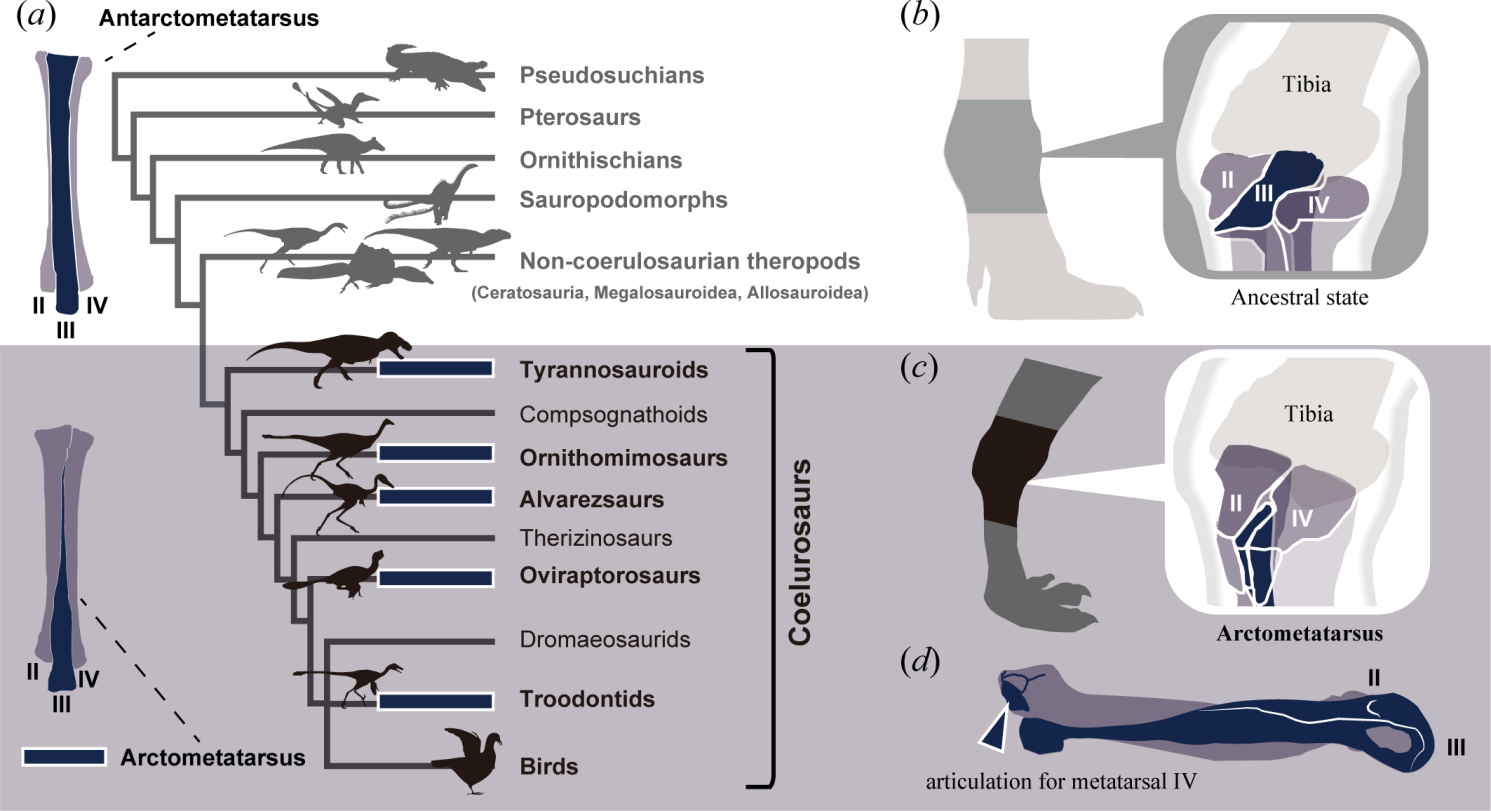
Evolution of the dinosaur ankle skeleton. (*a*) Phylogeny of archosaurs showing mapped foot morphological states. Above: the antarctometatarsus of *Kiyacursor longipes*, a noasaurid ceratosaur, in dorsal view (redrawn from [23]). Below: the arctometatarsus of *Gallimimus bullatus* (MPC-D 100/52) in dorsal view. (*b*) Ancestral and antarctometatarsalian arrangements of metatarsals in the ankle. (*c*) Arctometatarsalian metatarsal arrangements in the ankle. (*d*) Right foot of the tyrannosaurid *Gorgosaurus libratus* (UALVP 10) in sagittal cross-section, shown in articulation. Dark blue squares indicate independent evolutionary origins of the arctometatarsalian foot. Abbreviations: MPC, Institute of Paleontology of the Mongolian Academy of Sciences, Ulaanbaatar, Mongolia; UALVP, University of Alberta Laboratory for Vertebrate Paleontology, Edmonton, Alberta. Silhouettes were sourced from Phylopic (http://phylopic.org/) and modified with kind permission from artworks by Genya Masukawa (*Jaculinykus, Ornithomimus* and *Gobivenator*).

The fossil record shows several morphological modifications in theropod hind limbs, which are related to their locomotor ecologies [18–22]. Major non-avialan coelurosaur lineages possess a specialized foot structure, the arctometatarsus (figure 1*a*,*c*,*d*), which is likely well adapted for cursoriality and agility [24–26]. On the other hand, outside this group, another specialized foot form, the antarctometatarsus (figure 1*a*), is known to have evolved in noasaurid ceratosaurs (character 155 in the appendix of [27]). These theropods also tend to have longer distal hind limb segments compared to other theropods [20,22–24]. The proximal and distal portions of the arctometatarsalian metatarsal III are displaced plantarly and dorsally relative to metatarsals II and IV, respectively (figure 1*c*,*d*). Consequently, the arctometatarsalian metatarsal III is out of alignment with the adjacent metatarsals (metatarsals II and IV) in the ankle, while the metatarsals II-IV in ancestral and antarctometatarsalian states lie in the same plane (figure 1*b*). This metatarsal arrangement evolved independently in five coelurosaur lineages (figure 1*a*) and has been the focus of attention in studies of functional morphology and evolution [24,28–30]. However, the evolutionary history of arctometatarsus remains less understood. Fossil remains of coelurosaurian theropods with arctometatarsus are abundant in Upper Cretaceous strata [31–34], but sparse in Lower Cretaceous strata [35]. This pattern supports the hypothesis that this convergent evolution occurred throughout the mid-Cretaceous. Here, we examine temporal evolutionary patterns in the occurrences of cursorial traits such as the arctometatarsus and hind limb proportions.

## 2. Material and methods

All analyses were performed in R [36] using custom code. The custom script was written in its associated packages ‘ape’ [37], ‘ggplot2’ [38], ‘phytools’ [39], ‘strap’ [40], ‘geiger’ [41], ‘paleotree’ [42] and ‘nlme’ [43].

### 2.1. Phylogenetic framework

An informal supertree of 180 taxa (electronic supplementary material, figure S1), consisting of 158 theropods, eight non-theropod dinosaurs and 14 Mesozoic non-dinosaurian archosaurs, was assembled from numerous literature sources as follows: Choiniere *et al*. [44], Canale *et al.* [45], Currie and Evans [46], Funston *et al.* [47], Hattori *et al.* [48], Kobayashi *et al*. [49], Kubo and Kubo [50], Kubo *et al.* [9], Lee *et al.* [51], Macdonald and Currie [52], McFeeters *et al.* [53], Nesbitt *et al.* [32], Tsuihiji *et al.* [54], Xing *et al.* [55] and Zanno *et al.* [31]. We reconstructed a phylogeny using Mesquite 3.40 [56]. Polytomies, reflecting phylogenetic uncertainty, were resolved randomly, and branch lengths were calibrated according to taxon ages, smoothing zero-length branches by equally sharing duration from the immediately basal non-zero length branch [42]. The topology was edited as necessary for subsequent analyses using the drop.tip function in the R package ‘ape’ [37].

### 2.2. Statistical analysis

Measurements of three hind limb segments (femoral circumference and lengths of femur, tibia and metatarsus) are available for 105 non-avialan dinosaur taxa (electronic supplementary material, table S1). All measurements were obtained from direct measurements of specimens and the published literature [9,45,47,48,52,53,57]. In this study, the femur circumference was used as a proxy for body mass [58–60]. For specimens missing femoral midshaft circumference data, we used the regression equation for bipedal dinosaurs to estimate the shaft oval circumference [60]. Except for alvarezsaurids, metatarsal III was used for the total metatarsal length. In this group, the measurement extended from the proximal ends of metatarsals II and IV to the distal end of metatarsal III due to the absence of the proximal half of metatarsal III in most alvarezsaurid specimens.

We corrected for phylogenetic and allometric effects by using the residuals of phylogenetically corrected generalized least squares (PGLS) regressions of each variable against log_10_-transformed femur circumference. To investigate the evolutionary patterns in lengths of the tibia, metatarsus and distal hind limb segments ( = tibia + metatarsus), we performed PGLS regressions of these measurements against femur circumference and calculated residuals from these regressions (electronic supplementary material, figure S2). We used the non-parametric Kruskal–Wallis test on these residuals to test differences in them among foot morphologies. Phylogenetic generalized ANCOVA analyses were also conducted to compare differences between arctometatarsalian and non-arctometatarsalian taxa in residual variance in conjunction with the phylogenetic regression parameters. Ancestral state estimation was then conducted using the ace function of ape on PGLS residuals rescaled between 0 and 1. These trait values were treated as continuous characters to both qualitatively map on the theropod tree and to assess the fit of a variety of macroevolutionary models implemented in the R package ‘OUwie’ [61]. We used the corrected AICc to determine whether the preferred model was significantly better than the next-best model.

### 2.3. Date estimation of occurrences of cursorial traits

We applied the cal3 method of Bapst [62,63] to estimate the divergence dates of the nodes, where the arctometatarsalian pes and the elongation of the tibia and metatarsal lengths occurred. Five coelurosaur lineages, in which the arctometatarsalian pes evolved or increased in the proportional lengths of the tibia and metatarsus, were observed, including tyrannosauroids, ornithomimosaurs, alvarezsaurs, oviraptorosaurs and troodontids. These nodes are based on the ancestral state reconstruction of arctometatarsus (electronic supplementary material, figure S5) and the results of the analysis in the previous section.

The cal3 method requires *a priori* estimates of diversification and sampling rates to infer likely divergence dates under a birth-death-sampling model and operates in a similar manner to the fossilized birth–death process. Sampling and extinction estimates were obtained by stochastically sampling sets of congruent taxon ranges from the occurrence data via the function ‘seqTimeList’ in the R package paleotree. We calculated maximum-likelihood estimates of sampling and extinction rates using the resulting range frequency distributions [64] and used our extinction rate estimates as a proxy for speciation rate. To account for the uncertainty in these rate estimates, each cal3 tree was time-scaled with a different set of estimated rates. As the cal3 approach is stochastic, we applied it 1000 times and aggregated the results into distributions.

## 3. Results

### 3.1. Phylogenetic trends of lengths in the pelvic limb

**Figure 2.**
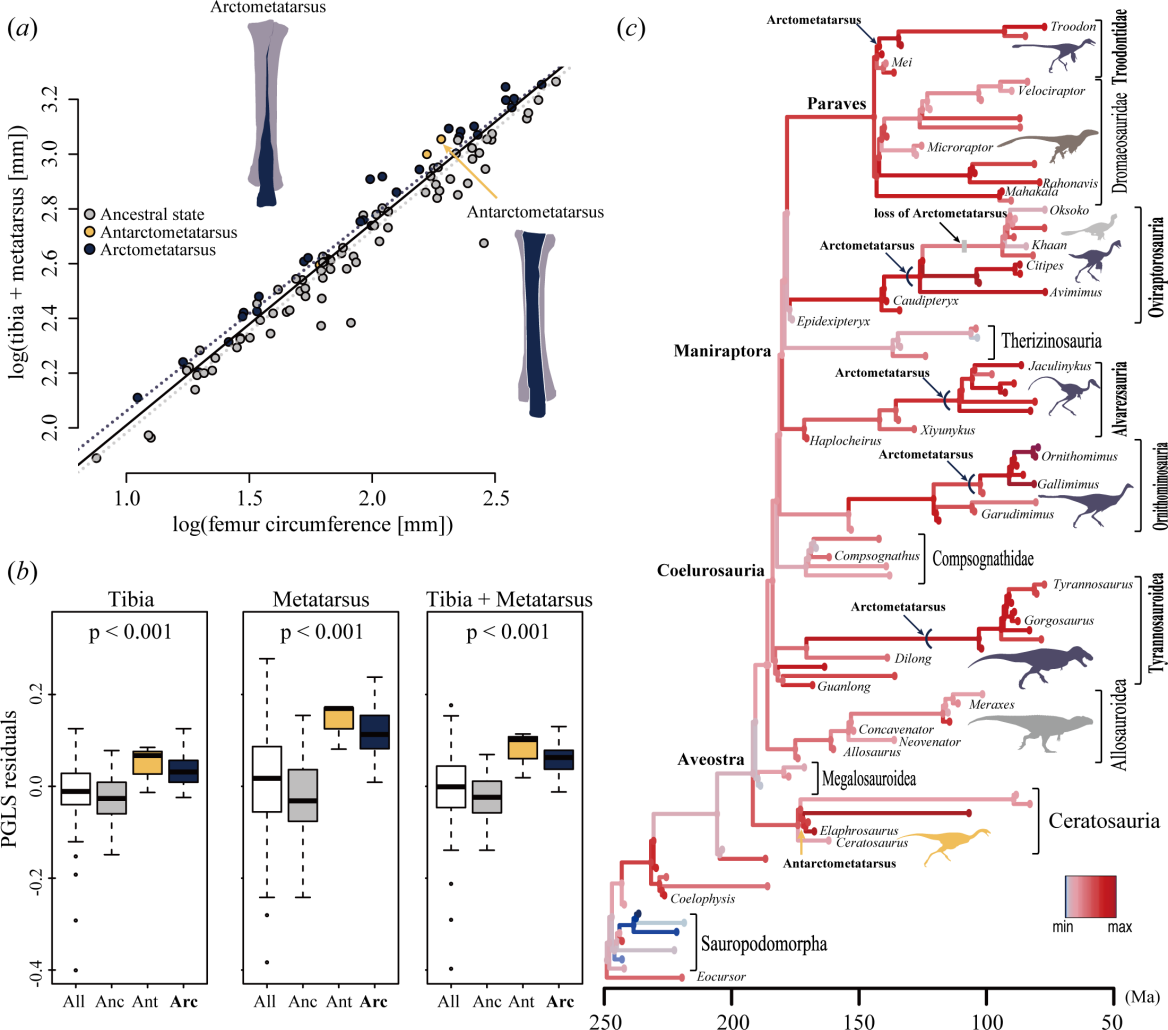
Proportional lengths of distal hind limb segments in theropod dinosaurs. (*a*) PGLS regression of log_10_-transformed distal hind limb length (= tibia + metatarsus) against log_10_-transformed femur circumference. (*b*) Residual plots from PGLS regressions of distal hind limb lengths (tibia, metatarsus, tibia + metatarsus) with specimens ordered according to foot morphologies (Anc, ancestral state; Ant, antarctometatarsus; Arc, arctometatarsus). (*c*) Temporal evolution of proportional length of distal hind limb segments (scaled between 0 and 1 residual distal hind limb length) mapped onto a time-scaled theropod phylogeny, with ancestral values estimated using the ace function of ape 5.3 [37]. The *x*-axis represents time in Ma.

To account for phylogenetic and allometric effects, we applied a phylogenetic generalized ANCOVA (phylANCOVA) model to clarify differences in the proportional lengths of hind limb segments (tibia, metatarsus and distal hind limb segments (= tibia + metatarsus)) among ancestral, antarctometatarsalian and arctometatarsalian states. The phylANCOVA analysis in metatarsus shows significant effects of foot morphologies on the log_10_-transformed variable of each hind limb segment (*p* < 0.05) after controlling for the also significant allometric effect of body size (log_10_-transformed femur circumference; table 1). Proportional lengths of hind limb segments among bipedal dinosaurs also show significant differences (*p* < 0.001) in the residuals of PGLS regressions of each variable against log_10_-transformed femur circumference across the three foot morphologies (figure 2*a*,*b*). Coelurosaurs with arctometatarsus and noasaurids with antarctometatarsus generally have proportionally longer tibia and metatarsus than other taxa. The proportional lengths of distal hind limb segments lengthened in nosaurid ceratosaurs and coelurosaurs including tyrannosauroids, ornithomimosaurs, alvarezsaurs, oviraptorosaurs and troodontids, and mostly coincide with occurrences of specialized foot morphologies (figure 2*b*,*c*; electronic supplementary material, figures S4 and S5). In ornithomimosaurs and oviraptorosaurs, the proportional length of the tibia and metatarsus increased in the clades of Ornithomimidae and Deinocheiridae, as well as in *Incisivosaurus* and its descendants including Caenagnathoidea, prior to the evolution of the arctometatarsus. Additionally, within caenagnathoid oviraptorosaurs, the proportional length of the metatarsus considerably shortened in oviraptorids with the ancestral metatarsal arrangement (figure 2*c*).

**Table 1.** Results of the phylogenetic ANCOVA model. PGLS regressions of log_10_-transformed femur circumference against independent variables for theropod dinosaurs. Abbreviations: Dhl, length of distal hind limb segments (tibia + metatarsus); fc, femur circumference.

independent variables		foot_morph	intercept	fc
~log_10_-transformed Dhl + foot_morph	AIC = −210.29	all	1.25	0.748
	*p*_fc_ < 0.001	ancestral	1.209	0.76
	*p*_ankle_morph_ = 0.067	**arctometatarsus**	1.351	0.711
~log_10_-transformed tibia + foot_morph	AIC = −136.02	all	1.05	0.756
	*p*_fc_ < 0.001	ancestral	1.02	0.762
	*p*_ankle_morph_ = 0.193	**arctometatarsus**	1.12	0.737
~log_10_-transformed metatarsus + foot_morph	AIC = −139.8	all	0.814	0.732
	*p*_fc_ < 0.001	ancestral	0.754	0.755
	*p*_ankle_morph_ = 0.045*	**arctometatarsus**	0.966	0.667

We used each set of the above PGLS residuals, rescaled between 0 and 1, as continuous characters for evolutionary model testing analysis using phylogenetic comparative methods. Model comparisons strongly support complex OU (= ‘Ornstein–Uhlenbeck’)-based models (OUMA) for each variable of these PGLS residuals. For the proportional length of distal hind limb segments (sum of tibia and metatarsus), the OUMA model was strongly supported with an AICc weight of 85.7%, values of *α* and *θ* were 0.06 and 0.82, respectively, for the arctometatarsus, and 0.06 and 0.93, for the antarctometatarsus, compared to 0.06 and 0.64 for the ancestral metatarsal arrangement (table 2). The phylogenetic half-lives ranged from 10.6 to 15.1 Myr for the arctometatarsus and 11.08 to 15.5 Myr for the antarctometatarsus. These results reveal that theropod taxa with foot specializations have higher trait optima (*θ*) compared with others, indicating constrained evolution, where trait optima play a more significant role than variance, predominantly shaping hind limb proportions in these taxa.

**Table 2.** Comparison of parameters across foot morphologies. OUMA model fits for PGLS residuals of distal hind limb segments. Abbreviations: Dhl, length of distal hind limb segments (tibia + metatarsus).

	parameter	ancestral state	arctometatarsus	antarctometatarsus
Dhl (= tibia + metatarsus)	*α*	0.069	0.064	0.062
	*σ* ^2^	0.003	0.003	0.003
	*θ*	0.646	0.823	0.926
tibia	*α*	0.074	0.069	0.064
	*σ* ^2^	0.004	0.004	0.004
	*θ*	0.714	0.842	0.958
metatarsus	*α*	0.049	0.045	0.044
	*σ* ^2^	0.003	0.003	0.003
	*θ*	0.553	0.809	0.905

### 3.2. The date estimation of cursorial trait evolution

**Figure 3.**
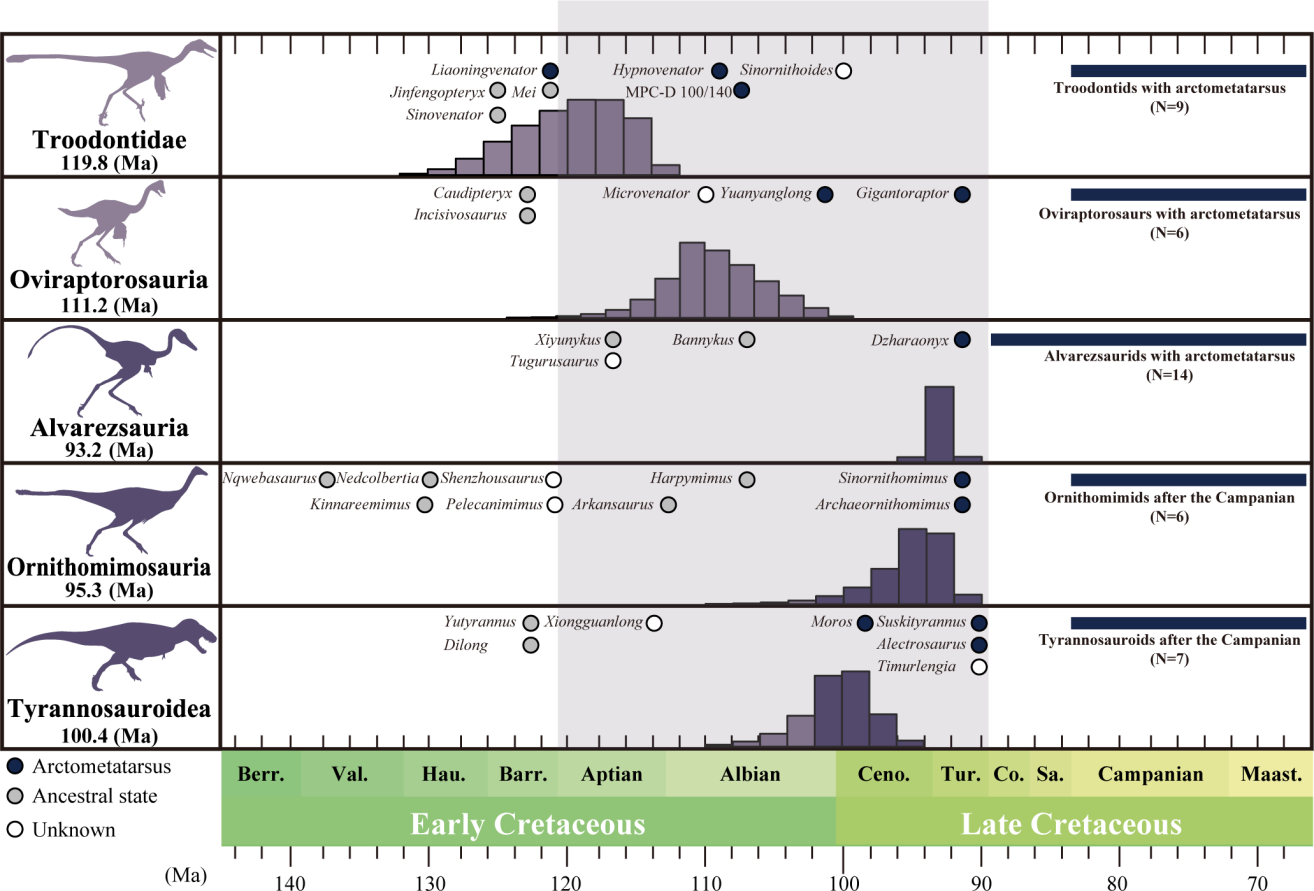
Probabilistic APT (‘*a posteriori*’ time-scaling) for key nodes in coelurosaur phylogeny illustrating the emergence of arctometatarsus. Coloured circles refer to the temporal occurrences of coelurosaurian theropods, with later-diverging members possessing the arctometatarsus during the mid-Cretaceous and early Cretaceous. Silhouettes were sourced from Phylopic (http://phylopic.org/) by Scott Hartman (*Tyrannosaurus*) and modified with kind permission from artworks by Genya Masukawa (*Gobivenator, Chirostenotes, Jaculinykus* and *Ornithomimus*). The horizontal axis represents time in Ma.

The results are summarized in figure 3. The arctometatarsus is estimated to have emerged in theropod lineages approximately 120−90 Ma (figure 3, electronic supplementary material, figure S2). Mean node age estimates for its evolution are 100.39 Ma in Tyrannosauroidea, 95.33 Ma in Ornithomimidae, 93.23 Ma in Alvarezsauridae except for *Alvarezsaurus*, 111.22 Ma in Oviraptorosauria and 119.77 Ma in Troodontidae. Excluding the oviraptorosaurs and troodontids, the occurrences of the arctometatarsalian foot in the remaining three lineages are nearly synchronous, occurring within an interval of less than 10 Myr. Additionally, except for Ornithomimidae and Oviraptorosauria, the appearance of the arctometatarsalian foot coincides with increases in the proportional length of the tibia and metatarsus, consistent with a previous study [24]. Mean node age estimates for the elongation of the tibia and metatarsus in Ornithomimosauria and Oviraptorosauria, where the elongation of the tibia and metatarsus are 118.8 and 129.1 Ma, respectively.

## 4. Discussion

### 4.1. Evolution of hind limb proportions and metatarsal arrangement

Our results revealed that proportional tibial and metatarsal lengths relative to their body mass lengthened independently in noasaurids and five coelurosaur clades such as tyrannosauroids, ornithomimosaurs, alvarezsaurids, oviraptorosaurs and troodontids (figure 2*a*–*c*). The arctometatarsalian arrangement also evolved independently in these five coelurosaur clades [24,29,30], whereas, outside Coelurosauria, the noasaurid metatarsus independently developed an antarctometarsalian state [23,27,65,66]. Comparative analysis of hind limb proportions in bipedal dinosaurs also demonstrates that the tibia, metatarsus and their combined lengths are significantly greater in arctometatarsus or antarctometatarsus than in taxa with the ancestral condition (figure 2*b*). Consistent with this, theropods with arctometatarsus tend to exhibit proportionally longer distal hind limb segments compared to those without as shown in previous studies [24,29,30]. Our phylANCOVA model also suggests a strong correlation between foot morphology and proportional lengths of distal hind limb segments.

In oviraptorosaurs, the arctometatarsus likely evolved prior to the common ancestor of Avimimidae and Caenagnathidae. A recently described oviraptorosaur, bearing an arctometataralian ankle morphology, has been recovered as the sister taxon to the clade of Avimimidae and Caenagnathoidea, further supporting this hypothesis [67]. On the other hand, the metatarsus in oviraptorids, later-diverging caenagnathoids, lacks the arctometatarsalian state and is proportionally shorter than that of caenagnathids and avimimids (figure 2*c*; electronic supplementary material, figures S4 and S5). This pattern of metatarsal evolution in oviraptorosaurs reinforces the evolutionary relationship between arctometatarsus and hind limb proportions, particularly proportional metatarsal length.

Phylogenetic distribution suggests that the arctometatarsus did not evolve outside Coelurosauria, despite the hind limb proportions of noasaurid ceratorsaurs being comparable to those of coelurosaurs with an arctometatarsus [23,66] (figure 2*b,c*). Instead, noasaurids evolved an antarctometatarsalian foot, characterized by a plantarly expanded and robust metatarsal III relative to metatarsals II and IV [23,27,66]. In the antarctometatarsus, metatarsals II–IV in the ankle joint remain in the same plane as the ancestral state, whereas in the arctometatarsus, metatarsal III is displaced plantarly relative to the adjacent metatarsals (figure 1*b*,*c*). These suggest that a phylogenetic constraint may have prevented modifications to metatarsal arrangement at the ankle joint outside Coelurosauria.

Our data further reveal a complex evolutionary history of the arctometatarsus. Despite a strong association between arctometatarsus and hind limb proportions, long proportional tibial and metatarsal lengths in ornithomimosaurs and oviraptorosaurs evolved before the emergence of the arctometatarsus (figure 2*c*). For example, *Kinnareemimus*, an early diverging ornithomimosaur, as well as early diverging troodontids and dromaeosaurids with proportionally long tibia and metatarsus, also possess another foot morphology, sub-arctometatarsus [30,68–70]. However, the ankle arrangement of sub-arctometatarsus is comparable to those of antarctometatarsalian and ancestral states, rather than arctometatarsus. Some paravian theropods without arctometatarsus, such as halszkaraptorine dromaeosaurids and early diverging avialans, exhibit proportionally long tibia and metatarsus, which are comparable to coelurosaurs with an arctometatarsus [22]. These findings imply greater complexity in the relationship between the evolutionary convergence of the arctometatarsus and hind limb proportions than previously inferred by comparative studies [24,30].

### 4.2. Temporal trend in cursorial trait evolution in Coelurosauria

Morphological modifications of the hind limb skeleton have proceeded significantly along the theropod lineage [18,19,57,71,72]. Among these adaptations, proportionally elongation of the tibia and metatarsus, as well as the evolution of the arctometatarsus, are considered key traits for cursorial lifestyles [20,22,24,28–30]. Fossil evidence for these cursorial traits is scarce prior to the mid-Cretaceous [35,68,73], but non-avian coelurosaurs adapted for cursoriality are abundant in the Upper Cretaceous of Asiamerica, consisting of North America and Asia [31–34]. Our analyses of date estimation, compiled for the fossil record of non-avialan coelurosaurs, demonstrated the following temporal patterns (figure 3): (i) the arctometatarsalian foot emerged through parallel evolution during the mid-Cretaceous (93–120 Ma) and (ii) proportional elongation of tibia and metatarsus occurred within this interval, except in Oviraptorosauria (129.1 Ma).

To date, the fossil record of some coelurosaurian lineages, such as alvarezsaurs, remains relatively scarce or fragmentary in Lower Cretaceous deposits [74–78]. The date estimation of cursorial traits in these lineages may eventually be revised earlier with future discoveries, given the temporal gaps in the fossil record. However, as metatarsal arrangements in coelurosaurs prior to the mid-Cretaceous were limited to ancestral or subarctometatarsalian states [30,68], our date estimations align with this pattern, highlighting the mid-Cretaceous emergences of the arctometatarsus and modifications in hind limb proportions (figure 3). These temporal patterns imply that coelurosaurian theropods during this time interval were likely subjected to strong ecological selection on hind limb morphology, adapted for cursoriality [79,80]. The arctometatarsal structure also offers greater durability than the ancestral condition, an adaptation that enhances cursoriality by enabling coelurosaurs to compensate for increased loading on the limb musculoskeletal systems [25,26,28]. Additionally, increases in the proportional lengths of distal hind limb segments may reflect strong selection pressures to maximize net energy efficiency [79,81–85]. Morphological adaptations in diet ecologies likely arose among insects, mammals and dinosaurs during the mid-Cretaceous [3,86–88], coinciding with the decline of non-coelurosaurian theropods in Asiamerica [31,89]. Although the connections between hind limb modifications in coelurosaurs and environmental changes in this time interval remain unclear, incorporating paleoenvironmental data may help to resolve this issue.

Outside Coelurosauria, noasaurid ceratosaurs from both Laurasian and Gondwanan continents also exhibit similar hind limb modifications from the Late Jurassic to Early Cretaceous [23,27,66], indicating that the timing of cursorial adaptations differs between coelurosaurian and non-coelurosaurian theropods. Moreover, coelurosaurs with a definitive arctometatarsalian foot have yet to be discovered in Gondwanan continents [69,90,91]. However, this absence does not necessarily imply that the evolutionary trend of enhanced cursorial performance in coelurosaur hind limb skeleton was geographically restricted. Instead, the limited fossil record of this group, such as paraves, alvarezsaurs and possibly megaraptors from Gondwanan continents, complicates interpretations of this evolutionary trend. Future discoveries may clarify whether these adaptations were geographically widespread or regionally constrained.

## 5. Conclusion

This study reveals the phylogenetic and temporal patterns of parallel evolution in the hind limbs of coelurosaurian theropods. Our data demonstrates a strong relationship between the arctometatarsus in coelurosaurs and long, proportionally tibial and metatarsal lengths. Temporal patterns of the evolution of the hind limb skeleton also show occurrences of arctometatarsus as well as changes in hind limb proportions in coelurosaurian theropods as parallel evolution during the mid-Cretaceous period, suggesting functional selection for cursoriality during this time interval. The evolutionary patterns of theropod hind limbs further highlight the parallel evolution of the arctometatarsus restricted to Coelurosauria, implying a phylogenetic constraint outside this clade that prevented its development. Indeed, outside Coelurosauria, noasaurid ceratosaurs lack the arctometatarsus but possess long proportional distal hindlimb segments comparable to coelurosaurs with this adaptation. Consequently, the genetic or developmental basis for the establishment of the arctometatarsus likely dates back to the origin of Coelurosauria.

## Data Availability

All raw data are available in the online version of this article including Supplementary Tables [92].
